# Deep Learning Reveals Key Immunosuppression Genes and Distinct Immunotypes in Periodontitis

**DOI:** 10.3389/fgene.2021.648329

**Published:** 2021-03-12

**Authors:** Wanchen Ning, Aneesha Acharya, Zhengyang Sun, Anthony Chukwunonso Ogbuehi, Cong Li, Shiting Hua, Qianhua Ou, Muhui Zeng, Xiangqiong Liu, Yupei Deng, Rainer Haak, Dirk Ziebolz, Gerhard Schmalz, George Pelekos, Yang Wang, Xianda Hu

**Affiliations:** ^1^Department of Conservative Dentistry and Periodontology, Ludwig-Maximilians-University of Munich, Munich, Germany; ^2^Dr. D. Y. Patil Dental College and Hospital, Dr. D. Y. Patil Vidyapeeth, Pune, India; ^3^Faculty of Dentistry, The University of Hong Kong, Hong Kong, China; ^4^Faculty of Mechanical Engineering, Chemnitz University of Technology, Chemnitz, Germany; ^5^Faculty of Physics, University of Münster, Münster, Germany; ^6^Zhujiang Hospital, Southern Medical University, Guangzhou, China; ^7^Laboratory of Cell and Molecular Biology, Beijing Tibetan Hospital, China Tibetology Research Center, Beijing, China; ^8^Department of Cariology, Endodontology and Periodontology, University of Leipzig, Leipzig, Germany; ^9^State Key Laboratory of Biocatalysis and Enzyme Engineering, Hubei Collaborative Innovation Center for Green Transformation of Bio-Resources, School of Life Sciences, Hubei University, Wuhan, China

**Keywords:** deep learning, autoencoder (AE), periodontitis, immunosuppression genes, therapeutic targets, bioinformatics

## Abstract

**Background:**

Periodontitis is a chronic immuno-inflammatory disease characterized by inflammatory destruction of tooth-supporting tissues. Its pathogenesis involves a dysregulated local host immune response that is ineffective in combating microbial challenges. An integrated investigation of genes involved in mediating immune response suppression in periodontitis, based on multiple studies, can reveal genes pivotal to periodontitis pathogenesis. Here, we aimed to apply a deep learning (DL)-based autoencoder (AE) for predicting immunosuppression genes involved in periodontitis by integrating multiples omics datasets.

**Methods:**

Two periodontitis-related GEO transcriptomic datasets (GSE16134 and GSE10334) and immunosuppression genes identified from DisGeNET and HisgAtlas were included. Immunosuppression genes related to periodontitis in GSE16134 were used as input to build an AE, to identify the top disease-representative immunosuppression gene features. Using K-means clustering and ANOVA, immune subtype labels were assigned to disease samples and a support vector machine (SVM) classifier was constructed. This classifier was applied to a validation set (Immunosuppression genes related to periodontitis in GSE10334) for predicting sample labels, evaluating the accuracy of the AE. In addition, differentially expressed genes (DEGs), signaling pathways, and transcription factors (TFs) involved in immunosuppression and periodontitis were determined with an array of bioinformatics analysis. Shared DEGs common to DEGs differentiating periodontitis from controls and those differentiating the immune subtypes were considered as the key immunosuppression genes in periodontitis.

**Results:**

We produced representative molecular features and identified two immune subtypes in periodontitis using an AE. Two subtypes were also predicted in the validation set with the SVM classifier. Three “master” immunosuppression genes, PECAM1, FCGR3A, and FOS were identified as candidates pivotal to immunosuppressive mechanisms in periodontitis. Six transcription factors, NFKB1, FOS, JUN, HIF1A, STAT5B, and STAT4, were identified as central to the TFs-DEGs interaction network. The two immune subtypes were distinct in terms of their regulating pathways.

**Conclusion:**

This study applied a DL-based AE for the first time to identify immune subtypes of periodontitis and pivotal immunosuppression genes that discriminated periodontitis from the healthy. Key signaling pathways and TF-target DEGs that putatively mediate immune suppression in periodontitis were identified. PECAM1, FCGR3A, and FOS emerged as high-value biomarkers and candidate therapeutic targets for periodontitis.

## Introduction

Periodontitis involves the inflammatory destruction of the supporting tissues of teeth. It involves a perturbed local host immune response that is ineffective in countering plaque biofilm microbiota (Meyle and Chapple, 2015). Innate and adaptive immunity work in tandem to counter the infectious challenge posed by oral microbiota, limit the spread of infection, and reestablish periodontal tissue homeostasis (Cekici et al., 2014). This delicately orchestrated process involves the actions of several immune regulatory cell types, including oral epithelial cells (Dutzan et al., 2016), neutrophils (Scott and Krauss, 2011), macrophages, dendritic cells (Zhou et al., 2019), B cells, and T cells (Gemmell et al., 2002). Regulatory T cells (Tregs) have particularly attracted much recent attention as they engender multiple suppressive mechanisms to inhibit various cells involved in innate and adaptive immunity. The role of Tregs in controlling periodontitis due to their immune-suppressive capabilities has been noted (Alvarez et al., 2018). Immune suppression demands the tandem action of multiple immunosuppression genes, several of which have been demonstrated in the context of periodontal pathology. These include programmed cell death 1 (PD1), PD-Ligand 1 (PD-L1) (Bailly, 2020), and Cytotoxic T-Lymphocyte Antigen4 (CTLA4) (Aoyagi et al., 2000), that function as immune checkpoint inhibitors to modulate B-cells, CD8+ T-cells, and CD4+ T-cells, which can amplify infection and promote tissue damage. Therefore, an immune checkpoint blockade has been proposed as a modality to manage periodontitis. However, existing reports have documented very few immunosuppression genes in the context of periodontitis. It is also recognized that immunosuppressive agents impose a risk for periodontal diseases, inducing gingival overgrowth or other alterations in periodontal tissues (Cota et al., 2010). Immunosuppressive medications for immune-related disorders such as rheumatoid arthritis or solid organ transplantation are associated with periodontal disease. However, the underlying molecular mechanisms remain unclear, and few genes have been implicated. For instance, specific Human Leukocyte Antigen (HLA)-DR1 genotype is documented to protect from gingival overgrowth induced by cyclosporine A (Cebeci et al., 1996). A more expansive understanding of immune suppression genes that are relevant to periodontal disease pathology can lead the identification of candidate genes and molecular pathways of significant potential translational value. Such data may enable the development of gene and targeted drug therapy for multiple periodontal diseases.

Experimental studies are limited by scale, incomplete or inaccurate existing databases, and the cost-intensive nature of molecular experiments, so approaches that can predict previously unidentified gene functions, enable gene function discovery, and automate the identification of inaccuracies can be very valuable (Chicco et al., 2014). Deep learning (DL) computational frameworks are capable of these. In this regard, an autoencoder (AE), is essentially a dimensionality reduction tool, as the “building block” of DL, comprises of a three-layered unsupervised artificial neural network that performs extraction of representative features (Lee et al., 2009; Wang et al., 2016). The AE has been implemented as a DL framework to predict survival in liver cancer (Chaudhary et al., 2018), breast cancer (Tan et al., 2014), head and neck squamous cell carcinoma (HNSCC) (Zhao et al., 2019), and when applied to RNA-seq data (Xiao et al., 2018) has shown value in generating key features from gene expression data that are linked to clinical outcomes.

To our knowledge, the present study is the first to integrate multi-omics data pertaining to immunosuppression genes in periodontitis using a DL-based AE combined with a support vector machine (SVM) classifier (Ju et al., 2015) confirmed in a validation set, along with an array of bioinformatic analysis, with an aim to identify the most significant immunosuppression genes relevant to the pathogenesis of periodontitis.

## Materials and Methods

### Study Design

**FIGURE 1:**
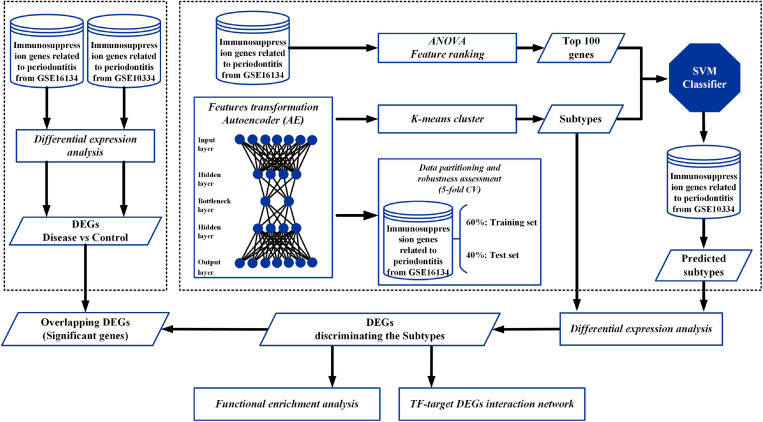
Overall workflow. The flowchart depicts the autoencoder (AE) architecture and workflow combining deep learning (DL) techniques to identify key immunosuppression genes in periodontitis. Immunosuppression genes related to periodontitis from GSE16134 were applied as input features for an AE. The new transformed features in the bottleneck layer of the AE were clustered into different subtypes using K-mean clustering. Then, based on the clustering labels, we selected the top 100 most related genes from GSE16134 based on ANOVA *F* values. The input dataset was split at a 60%/40% ratio (training set/test set) to assess the robustness of the AE, using a 5-fold CV. Subsequently, based on the above labels of GSE16134, an SVM classifier was built and further applied for prediction in a validation set (GSE10334). To explore the biological roles of the different identified subtypes, differentially expressed genes (DEGs) and transcription factors (TFs), differential expression analysis, functional enrichment analysis, and construction of TF-target DEGs interaction network were, respectively, applied. Eventually, to identify the immunosuppression genes that might be most pertinent to periodontitis, the overlapping DEGs among the DEGs discriminating disease (periodontitis) and controls and DEGs discriminating the subtypes classified with the AE and SVM models were determined.

An overview of the workflow of this study is depicted in Figure 1. In brief, two cohorts of periodontitis datasets (GSE16134 and GSE10334) and immunosuppression genes were included. First, immunosuppression genes related to periodontitis from GSE16134 were identified and applied as input features to build an AE model. Second, each of the new transformed features in the bottleneck layer of the AE was clustered into different subgroups using K-mean clustering. In addition, based on the clustering labels, we selected the top 100 most related genes from GSE16134 based on ANOVA *F* values. Data partitioning of the inferring samples of GSE16134 was applied to assess the robustness of the AE, using a 5-fold CV. The samples were randomly split into 5 folds, 3 of which were used as the training set (60%) and the remaining 2 (40%) as the test set. Thereafter, based on the clustering results and the top 100 genes of GSE16134, a SVM classifier was built with a 5-fold CV to identify the optimal hyperparameters, and a validation set (immunosuppression genes related to periodontitis in GSE10334) was applied for SVM to predict the subtypes. To explore the biological roles of the different identified subtypes, differentially expressed genes (DEGs) and transcription factors (TFs), differential expression analysis, functional enrichment analysis, and construction of TF-target DEGs interaction network were, respectively, applied. Finally, to identify the immunosuppression genes that might be most pertinent to periodontitis, the overlapping DEGs among the DEGs discriminating periodontitis and controls and DEGs discriminating the subtypes classified with the DL-based model were determined.

### Pre-processing of the Dataset

Transcriptomic data from gingival tissue samples affected with periodontitis and the corresponding controls (GSE16134 and GSE10334) were obtained from the Gene Expression Omnibus (GEO) database of NCBI^1^. Detailed information of the two datasets is listed in Table 1. Immunosuppression genes were obtained from databases DisGeNET^2^ and HisgAtlas^3^. From these obtained genes, 1,207 immunosuppression genes related to periodontitis were extracted. Next, the two datasets were stacked, and 1,181 immunosuppression genes’ expression profiles were found matching in the two datasets. Subsequently, the two datasets were standardized using the “scale” function in R, setting the parameters as (scale = TRUE and center = FALSE).

**TABLE 1:** Summary of periodontitis related GEO datasets used in this study.

**Data**	**GPL (General public license)**	**Gene**	**Sample control**	**Sample case**
GSE16134	GPL570	24441	69	241
GSE10334	GPL570	24441	64	183

### Features Transformation

Immunosuppression gene expression profiles of 241 disease samples in the GSE16134 dataset were selected as the input for the AE. The re-coding of the DL algorithms was performed using the Python library “Keras^4^”. An AE is a three-layered neural network consisting of input, hidden, and output layers (Wang et al., 2016), and here an AE with three hidden layers was implemented with 200, 100, and 200 nodes per layer each. One hundred nodes produced by the bottleneck layer were regarded as the new compressed representative features of the data. In accordance with previous research, the AE was set up using the following equations (Chaudhary et al., 2018).

$$y=f_{i}\left(x\right)=tanh\left(w_{i}.x+b_{i}\right)$$

x′=F1→k⁢(x)=f1o⁢…o⁢fk-1oo⁢fk⁢(x)

logloss⁢(x,x′)=∑k=1d(xk⁢l⁢o⁢g⁢(x′k)+(1-xk)⁢l⁢o⁢g⁢(1-x′k))

$$L\,\left(x,x^{\prime }\right)=\text{logloss}\,\left(x,x^{\prime }\right)+\sum_{i=1}^{k}\left(\partial_{w}\,{||W_{i}||}_{1}+\partial_{a}\,{||F_{1\to i}\,\left(x\right)||}_{2}^{2}\right)$$

To control overfitting, the penalty values αα and αw (the activity regularizer of layer output) were set to 0.00002 and 0.00001. In addition, the AE was trained using the gradient descent algorithm with 20 epochs and 50% dropout. Here, an epoch is an iteration that indicates the number of passes of the entire training dataset, while the size 20 is one of the appropriate training cycles calculated in the evaluation of the model.

### K-Means Clustering to Identify Subtypes of Immunosuppression Genes in Periodontitis

The 100 nodes from the bottleneck-hidden layer were considered as new features for the analysis and were clustered with the K-means algorithm. The optimal number of clusters was determined based on two metrics: Silhouette index (Rousseeuw, 1987) and Calinski–Harabasz index (Calinski and Harabasz, 1974), using scikit-learn package (Pedregosa et al., 2011).

### Comparison of AE With PCA Based Clustering

Principal component analysis (PCA), a conventional dimension reduction approach was applied to compare with the AE performance (Chaudhary et al., 2018). The same number (100) of the principal components were set as the features in the bottleneck layer and clustering performances of AE and PCA were evaluated using the Silhouette index (Rousseeuw, 1987).

### Data Partitioning and Robustness Assessment

Data partitioning of the inferring samples of GSE16134 was done to assess the robustness of the model, using a cross-validation (CV)–like procedure, as described in earlier reports (Chaudhary et al., 2018; Zhao et al., 2019). First, the samples were randomly split into 5 folds, 3 of which were used as the training set (60%) and the remaining 2 (40%) as the test set. Using this CV approach,10 new combinations (folds) were obtained. In each, a distinct AE and a classifier were constructed in each training fold and were used for predicting the labels in the test set. Eventually, category labels were inferred using an AE based on all the samples, and these labels were used for predicting labels of the validation dataset.

### Supervised Classification

First, the obtained features from GSE16134 were standardized with the “scale” function in R, setting the scale as (center = TRUE and scale = TRUE). Then, the top 100 “most relevant” immunosuppression genes in GSE16134 were selected based on the clustering labels and analysis of variance (ANOVA) *F* values. Since the top 100 genes were also present in GSE10334 dataset, a complementation test for missing genes was not conducted. Subsequently, based on the labels assigned using GSE16134, a SVM classifier was built and further applied for prediction in a validation set (GSE10334). The “scikit-learn” package (Pedregosa et al., 2011) was used to perform a grid search for the identification of the optimal hyperparameters for the SVM model using a 5-fold CV.

### Evaluation of the SVM Classifier

Accuracy and area under the curve (AUC) were selected as two metrics to evaluate the performance of the SVM classifier. The percentage of accuracy was calculated as: Accuracy (%) = Predict number / Test number. A receiver operating characteristic (ROC) curve was plotted for the model using the “pROC” (Robin et al., 2011) and the “ggplot2” packages in R^5^. The AUC is the area under the ROC curve, where an AUC value above 70% is considered acceptable (Mandrekar, 2010).

### Differential Expression Analysis

Differential expression analysis was performed for each of the datasets (GSE16134 and GSE10334), to identify genes discriminating between the disease and control samples, using the “Linear Models for Microarray data” (“limma”) package in R (Ritchie et al., 2015). Genes with *P* value < 0.05, and |log FC| ≥ 1 was selected as differentially expressed genes (DEGs). The DEGs with Log FC ≥ 1 was defined as up-regulated DEGs, while the DEGs with log FC ≤ −1 were defined as down-regulated DEGs.

Differential expression analysis was also similarly conducted for the classified subtypes. Here, genes with *P* value < 0.05, and |log FC| ≥ 0.05 were selected as DEGs; The DEGs with Log FC ≤ 0.05 were defined as up-regulated DEGs, while the DEGs with log FC ≤ −0.05 were defined as down-regulated DEGs.

To identify the most critical immunosuppression genes in periodontitis, the DEGs discriminating disease and control samples that overlapped with DEGs discriminating the different subtypes were identified and visualized using a Venn diagram. To evaluate the performance of each such identified gene, a ROC curve was plotted as described earlier.

### Functional Enrichment Analysis

The DEGs overlapping in the two datasets (GSE16134 and GSE10334) were identified using the “ClusterProfiler” package in R (Yu et al., 2012). The functions of these DEGs were explored by investigating their enriched Gene Ontology (GO) terms, particularly biological processes (BPs) and Kyoto Encyclopedia of Genes and Genomes (KEGG) pathways. The GO/BP terms and KEGG pathways with *P* value < 0.05 were regarded as significant functions. The top 30 of the enriched GO/BPs and pathways were chosen to be visualized in a bar plot.

In addition, KEGG pathway analysis was applied to determine the characteristics of different subtypes in GSE16134 and GSE10334 each. KEGG pathways with *P* value < 0.05 were regarded as significant functions. The top 20 of the enriched pathways were listed and visualized using the heatmap function in R (Galili et al., 2017).

### Construction of TF-Target DEGs Interaction Network

TF-target gene interaction pairs were downloaded from multiple databases, including TRRUST^6^, cGRNB^7^, HTRIdb^8^, ORTI^9^, and TRANSFAC^10^. The TFs targeting DEGs overlapping in the two datasets (GSE16134 and GSE10334) were extracted and used for constructing the TFs-target DEGs interaction network. The network was visualized using Cytoscape (Version 3.7.2) (Shannon et al., 2003), and the topological characteristics of the nodes in the TF-target gene network were determined.

## Results

### Identification of Two Subtypes of Immunosuppression Genes in GSE16134 by AE

**FIGURE 2:**
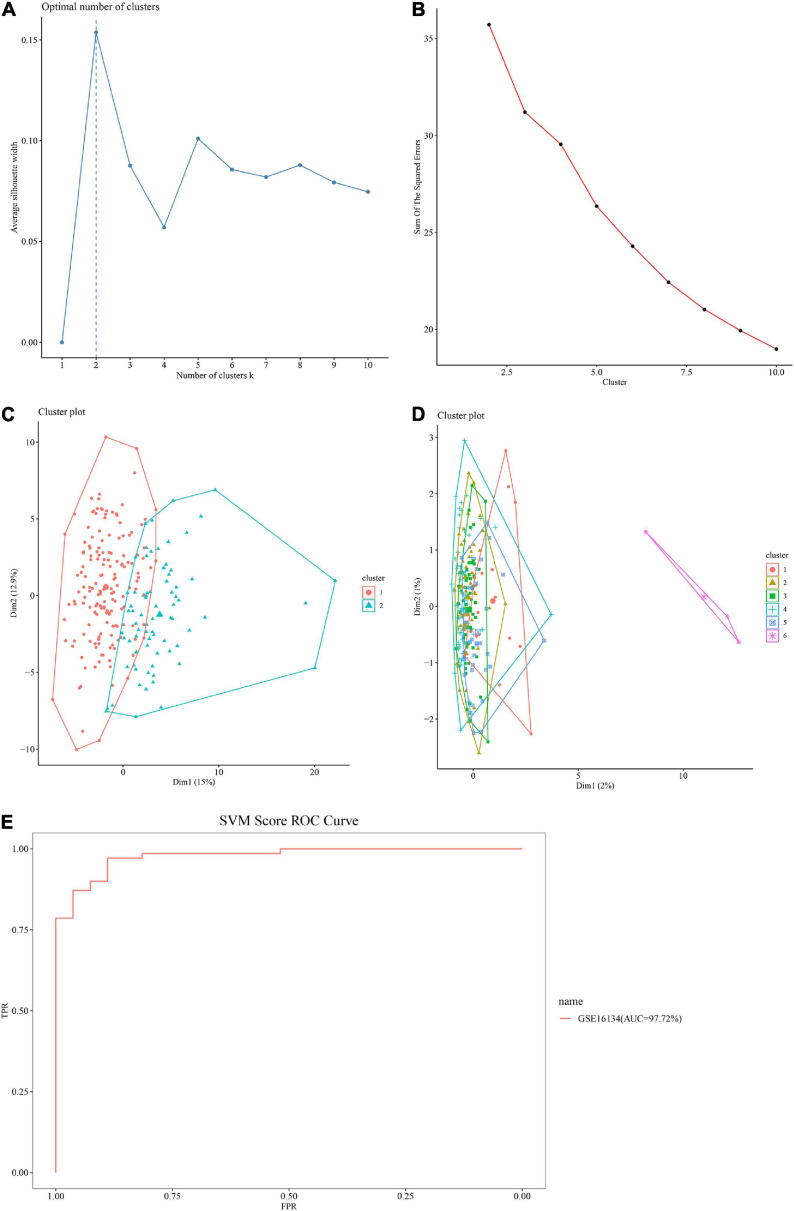
Performance of the autoencoder (AE) and support vector machine (SVM) model. **(A)** Clustering results using the Silhouette index. Horizontal axis: Average silhouette width; Vertical axis: Number of clusters k. The optimal number of clusters is 2. **(B)** Clustering outcomes using Calinski–Harabasz criterion. Horizontal axis: Sum of the squared errors; Vertical axis: Number of clusters k. The optimal number of clusters is 2. **(C,D)** Comparison of AE with principal component analysis (PCA) based clustering. **(C)** The performance of AE based on Silhouette index. The optimum cluster number using AE is 2. Dim = dimensions. **(D)** The performance of PCA based on Silhouette index. The optimum cluster number using PCA is 6. Dim = dimensions. **(E)** Receiver operating characteristic (ROC) curve of the SVM model. Horizontal axis: false discovery rate (FDR); Vertical axis: true positive rate (TPR). The area under the curve (AUC) value of the GSE16134 test set is 97.72%.

The optimal number of clusters was determined based on two metrics: Silhouette index (Figure 2A) and Calinski–Harabasz index (Figure 2B). The value of the silhouette coefficient is between [−1, 1] and the score near 1 indicates a highly dense clustering. When *k* = 2, the average silhouette width was nearest to 1 (Figure 2A). Using Calinski–Harabasz index, better performance of clustering depends on a higher score and at *k* = 2, the score (sum of the squared errors) was the highest (Figure 2B). Therefore, the genes were clustered into two subtypes, defined as S1 and S2.

### The AE Performed Better Compared to PCA

The performance of the AE was compared to that of PCA based clustering using Silhouette index. While two optimal clusters were extracted by AE (Figure 2C), six optimal clusters were extracted using PCA (Figure 2D), indicating that the difference between PCA transformed features was minimal, and it was difficult to cluster them effectively. Furthermore, the PCA landing points were concentrated in one zone, and the division was not clear. Therefore, the AE emerged as more effective and accurate in clustering features.

### SVM Model and Its Validation

Using a 5-fold CV, the input dataset (immunosuppression genes related to periodontitis from GSE16134) were split at a 60%/40% ratio for the training set and testing set. The SVM model presented an accuracy of 92.78% (Table 2), and the AUC score at 97.72%, above 90% (Figure 2E), supporting the model was efficient in distinguishing between classes and thus reliable in predicting significant immunosuppression genes in the GSE10334 dataset (Mandrekar, 2010).

**TABLE 2:** Classifier performance outcomes of SVM.

**GSE16134**			
	**Test**	**Predict**	**Accuracy (%)**

Cluster 1	27	21	
Cluster 2	70	69	
Total	97	90	92.78%

### DEGs Involved in Immunosuppression and Periodontitis

**FIGURE 3:**
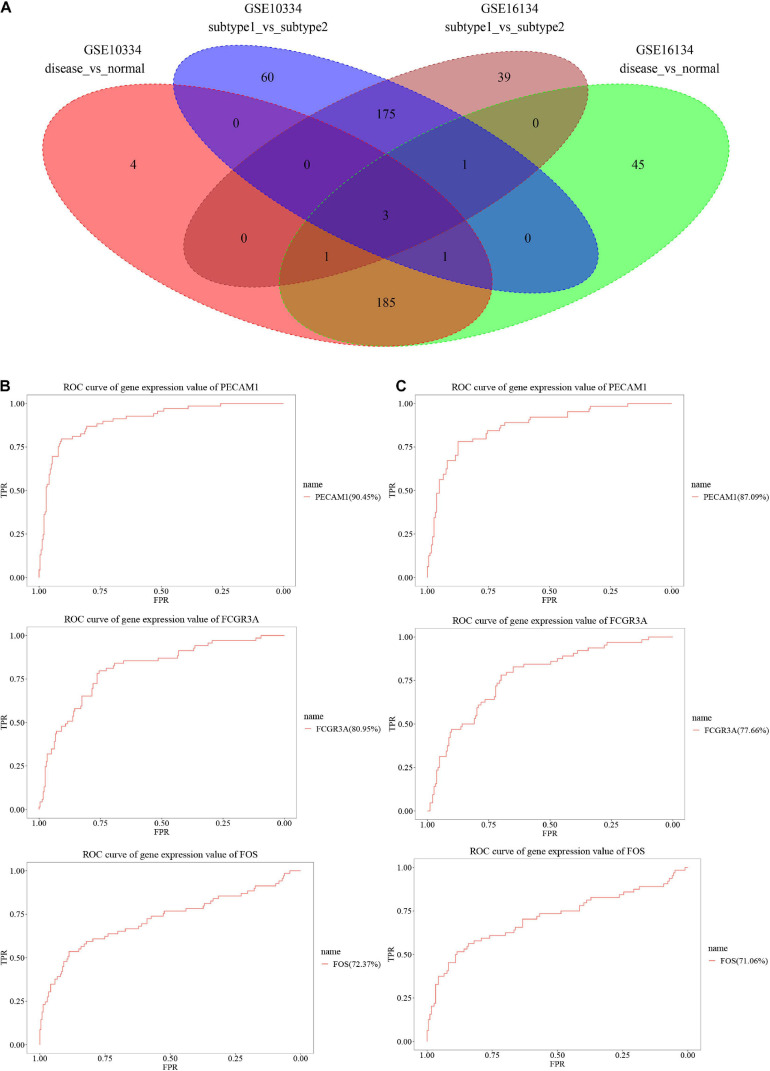
Identification of the significant DEGs. **(A)** Intersection of DEGs discriminating sample type (disease vs. normal) (236 DEGs from GSE16134 and 194 DEGs from GSE10334) and DEGs of the disease samples classified into subtypes (subtype 1 vs. subtype 2) (219 DEGs from GSE16134 and 240 DEGs from GSE10334). **(B,C)** ROC curve of three significant genes (PECAM1, FCGR3A, and FOS) in GSE16134 **(B)** and GSE10334 **(C)**. Horizontal axis: false discovery rate (FDR); Vertical axis: true positive rate (TPR).

Differential expression analysis was applied to the disease and control samples, as well as the two classified subtypes. A total of 236 DEGs consisting of 48 down-regulated DEGs and 188 up-regulated DEGs were identified from the GSE16134 dataset, while a total of 194 DEGs consisting of 42 down-regulated DEGs and 152 up-regulated DEGs were identified from the GSE10334 dataset (Table 3). For discriminating the designated subtype labels, a total of 219 DEGs consisting of 85 down-regulated DEGs and 134 up-regulated DEGs were identified in the GSE16134, while a total of 240 DEGs consisting of 95 down-regulated DEGs and 145 up-regulated DEGs were identified in the GSE10334 dataset (Table 4). As shown in the Venn diagram (Figure 3A), three significant DEGs, Platelet Endothelial Cell Adhesion Molecule (PECAM) 1, Fc Gamma Receptor (FCGR) 3A, and FOS were found intersecting and considered as potentially most robust immunosuppression genes related to periodontitis. Each of the three DEGs has an acceptable performance, with an AUC value above 70%, listed in Table 5. The ROC curves of the three genes from GSE16134 and GSE10334 are shown in Figures 3B,C, respectively.

**TABLE 3:** Outcome of differential gene expression analysis for datasets GSE16134 and GSE10334.

**Data (Disease vs. Normal)**	**DEG (Up)**	**DEG (Down)**	**Total**	**Log FC Abs**	***P* value**
GSE16134	188	48	236	>1	<0.05
GSE10334	152	42	194	>1	<0.05

**TABLE 4:** Differential expression analysis applied to disease samples based on identified subtypes.

**Data (Subtype1 vs. Subtype 2)**	**DEG (Up)**	**DEG (Down)**	**Total**	**Log FC Abs**	***P* value**
GSE16134	134	85	219	>0.05	<0.05
GSE10334	145	95	240	>0.05	<0.05

**TABLE 5:** AUC values of the three most significant genes.

**Gene**	**GSE10334_ROC_AUC (%)**	**GSE16134_ROC_AUC (%)**	**Mean (%)**
PECAM1	87.09	90.45	88.77
FCGR3A	77.66	80.95	79.31
FOS	71.06	72.37	71.72

### Functional Terms Enriched Among the DEGs

**FIGURE 4:**
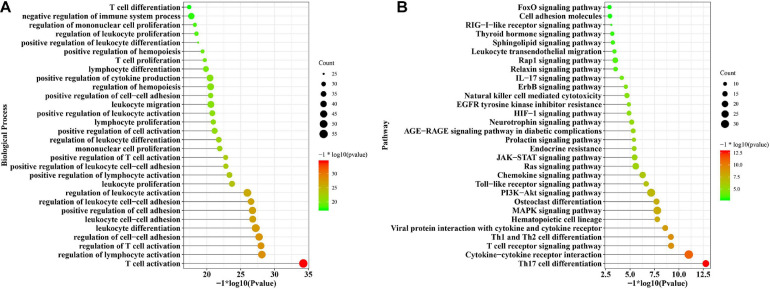
The functional enrichment analysis of the overlapping DEGs common to the two datasets (GSE16134 and GSE10334). **(A)** The significantly enriched biological processes of the overlapped DEGs; **(B)** The significantly enriched signaling pathways of the overlapped DEGs.

Significantly enriched biological processes and signaling pathways related to the immunosuppressive DEGs were identified from those overlapping between GSE16134 and GSE10334. The immunosuppressive DEGs involved in periodontitis were implicated in biological processes, including T cell activation, regulation of lymphocyte activation, regulation of T cell activation, regulation of cell-cell adhesion, and leukocyte cell-cell adhesion (Figure 4A). The immune activities were mainly regulated by Th17 cell differentiation, cytokine-cytokine receptor interaction, T cell receptor signaling pathway, Th1 and Th2 cell differentiation, Mitogen-activated Protein Kinase (MAPK) signaling pathway, osteoclast differentiation, and Phosphatidylinositol 3-Kinase (PI3K)-Protein Kinase B (Akt) signaling pathway (Figure 4B).

**FIGURE 5:**
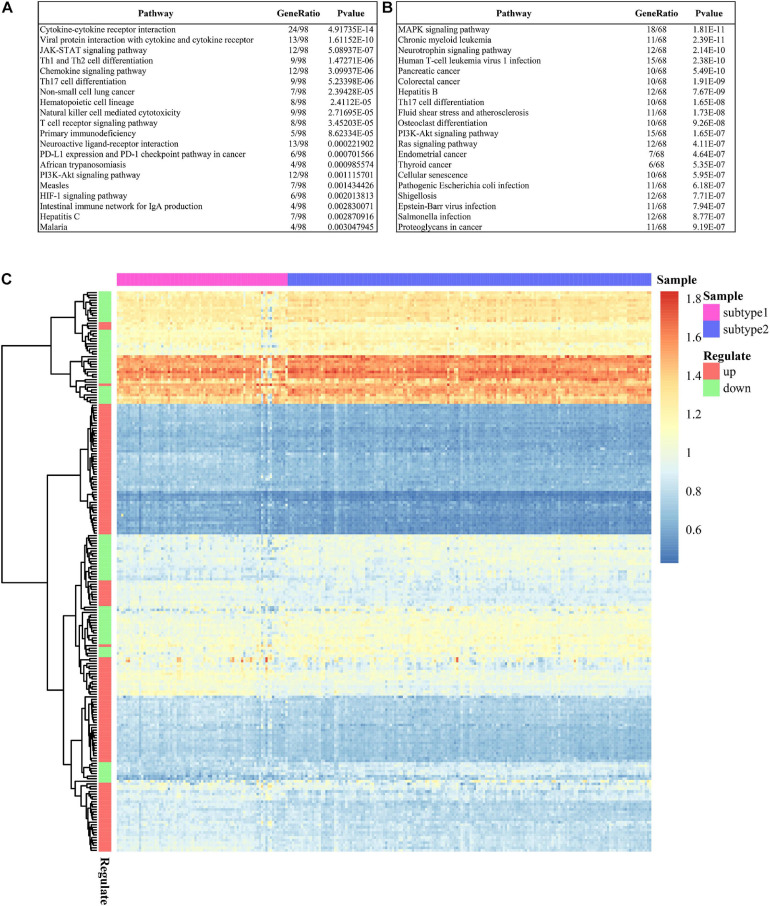
Pathways enriched in the DEGs characterizing the two subtypes in GSE16134. **(A)** Top 20 enriched signaling pathways of DEGs in subtype 1. **(B)** Top 20 enriched signaling pathways of DEGs in subtype 2. **(C)** Heatmap shows the enriched signaling pathways of DEGs in the two subtypes.

**FIGURE 6:**
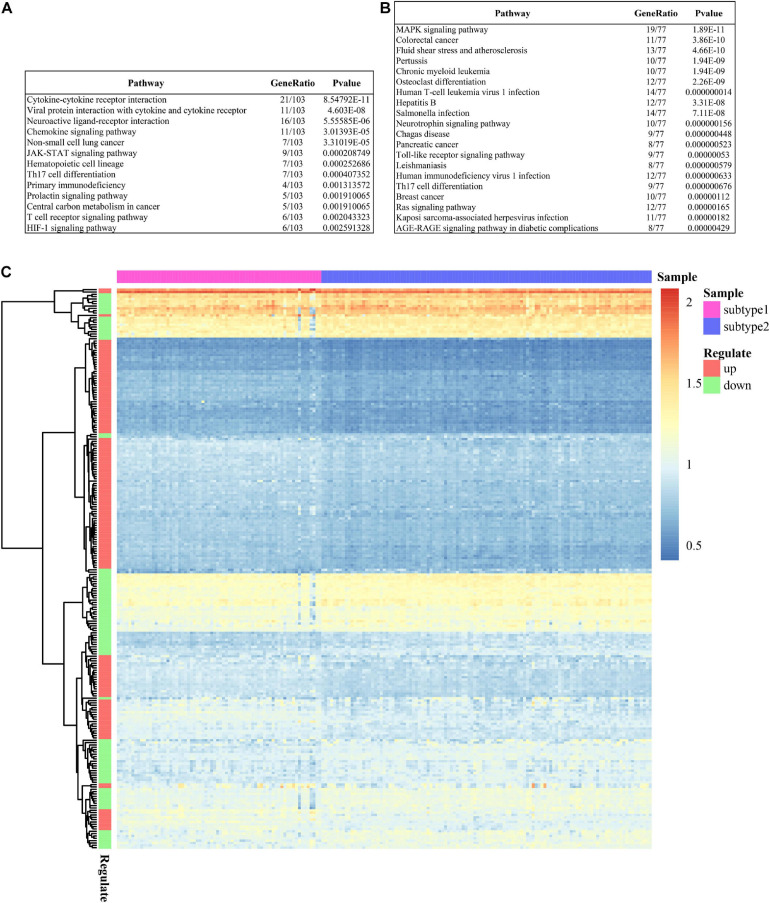
Pathways enriched in the DEGs characterizing the two subtypes in GSE10334. **(A)** Top 20 enriched signaling pathways of DEGs in subtype1. **(B)** Top 20 enriched signaling pathways of DEGs in subtype 2. **(C)** Heatmap shows the enriched signaling pathways of DEGs in the two subtypes.

Most pathways of the two subtypes were evident as distinct in GSE16134 (Figure 5), indicating significant differences between the two subtypes in terms of immunosuppressive activities in periodontitis. This difference was also detected between the two predicted subtypes in GSE10334 (Figure 6). Specifically, subtype S1 of immunosuppressive DEGs in periodontitis from both GSE16134 (Figure 5A) and GSE10334 (Figure 6A) was mainly enriched in cytokine-cytokine receptor interaction, chemokine signaling pathway, Janus kinase (JAK)- Signal Transducer and Activator of Transcription Protein (STAT) signaling pathway, Hypoxia-inducible Factor (HIF)-1 signaling pathway, and T cell receptor signaling pathway. Of note, subtype S1 from GSE16134 was also enriched in PD-L1 expression and PD-1 checkpoint pathway in cancer (Figure 5A). Whereas subtype S2 was mainly associated with MAPK signaling pathway, osteoclast differentiation, and infection of virus and *E. coli* bacteria (Figures 5B, 6B).

### Identification of Hub Transcription Factors That Targeted DEGs

**FIGURE 7:**
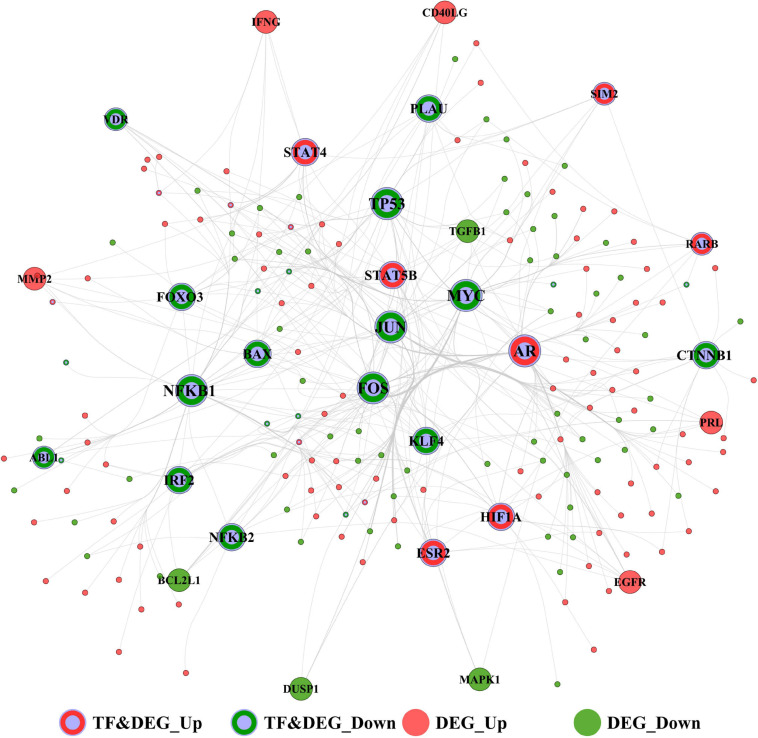
The transcription factor (TF)-target interaction network of GSE16134 and GSE10334 involved in immunosuppression and periodontitis. Top 30 TFs were visualized in the network. Red and gray dots: up-regulated TF and DEG; Green and gray dots: down-regulated TF and DEG; Red dots: up-regulated DEG; Green dots: down-regulated DEG.

The TFs-target DEGs interaction network of the immunosuppression genes in periodontitis is shown in Figure 7, consisting of 197 nodes and 447 edges. Top 30 TFs (Table 6) with the highest degree were considered to represent those most critical to this network. Of these, the top 10 TFs in the network were determined as the hubs, including Androgen Receptor (AR), Hypoxia-inducible Factor (HIF)1A, Signal Transducer and Activator of Transcription Protein (STAT) 5B, and STAT4, which were not only TFs but also up-regulated DEGs, and Nuclear Factor Kappa B Subunit 1 (NFKB1), MYC, JUN, Tumor Protein (TP)53, FOS, and Forkhead Box (FOX) O3, which were not only TFs but also down-regulated DEGs.

**TABLE 6:** The topological characteristics of the top 30 nodes in the TF-target interaction network.

**Name**	**Label**	**Degree**	**Average Shortest Path Length**	**Betweenness Centrality**	**Closeness Centrality**	**Clustering Coefficient**	**Topological Coefficient**
AR	TF&DEG_Up	87	1.6327	0.4063	0.6125	0.0270	0.0351
NFKB1	TF&DEG_Down	62	1.7143	0.2926	0.5833	0.0518	0.0457
MYC	TF&DEG_Down	56	1.7398	0.2342	0.5748	0.0610	0.0497
JUN	TF&DEG_Down	49	1.8163	0.1276	0.5506	0.0859	0.0541
TP53	TF&DEG_Down	41	2.0816	0.0906	0.4804	0.0634	0.0603
FOS	TF&DEG_Down	40	1.8776	0.0975	0.5326	0.1077	0.0670
HIF1A	TF&DEG_Up	17	2.0051	0.0433	0.4987	0.1985	0.1076
STAT5B	TF&DEG_Up	16	2.5408	0.0063	0.3936	0.0917	0.1517
FOXO3	TF&DEG_Down	15	2.3418	0.0175	0.4270	0.2000	0.1202
STAT4	TF&DEG_Up	15	2.0459	0.0329	0.4888	0.2190	0.1298
CTNNB1	TF&DEG_Down	14	2.1837	0.0514	0.4579	0.1648	0.1186
BAX	TF&DEG_Down	13	2.2857	0.0264	0.4375	0.1795	0.1348
KLF4	TF&DEG_Down	13	1.9949	0.0268	0.5013	0.2821	0.1560
IRF2	TF&DEG_Down	13	2.5918	0.0283	0.3858	0.0385	0.1110
ESR2	TF&DEG_Up	11	2.2092	0.0060	0.4527	0.3455	0.1706
NFKB2	TF&DEG_Down	10	2.3827	0.0182	0.4197	0.2889	0.1806
PLAU	TF&DEG_Down	10	2.4694	0.0140	0.4050	0.1556	0.1538
EGFR	DEG_Up	9	2.1327	0.0037	0.4689	0.4167	0.2059
VDR	TF&DEG_Down	9	2.4388	0.0030	0.4100	0.2500	0.1993
RARB	TF&DEG_Up	8	2.2245	0.0083	0.4495	0.3571	0.2083
BCL2L1	DEG_Down	7	2.1531	0.0021	0.4645	0.6190	0.2706
SIM2	TF&DEG_Up	7	2.2245	0.0045	0.4495	0.2857	0.2351
ABL1	TF&DEG_Down	6	2.2959	0.0029	0.4356	0.3333	0.2561
TGFB1	DEG_Down	6	2.0816	0.0005	0.4804	0.8667	0.3209
PRL	DEG_Up	6	2.4286	0.0014	0.4118	0.4000	0.2434
CD40LG	DEG_Up	6	2.4694	0.0031	0.4050	0.4000	0.2508
IFNG	DEG_Up	6	2.5918	0.0013	0.3858	0.3333	0.2405
MMP2	DEG_Up	6	2.4031	0.0003	0.4161	0.8000	0.2670
DUSP1	DEG_Down	5	2.2245	0.0022	0.4495	0.4000	0.3270
MAPK1	DEG_Down	5	2.3061	0.0009	0.4336	0.5000	0.3090

## Discussion

In this study, we used a DL-based algorithm, the AE, for identifying the pivotal immunosuppression genes relevant to periodontitis. With this approach, we re-constructed multi-omics data and produced representative molecular features grouped into two immune subtypes and then built an SVM model based on these, which was confirmed using a validation set. Besides, significant pathways and TF-target DEGs involved in immunosuppression during periodontitis were identified. Notably, we identified the key characteristics of two immune subtypes of periodontitis. We also identified three “master” immunosuppression genes, PECAM1, FCGR3A, and FOS, as candidate genes central to immune suppressive pathogenic mechanisms in periodontitis.

An AE-based DL approach has demonstrated high efficiency and accuracy in predicting biomarker genes for lung cancer, breast cancer, and HNSCC (Xiao et al., 2018). Akin to these studies, CV results indicated this approach was robust in classifying patients into two subgroups. Furthermore, the AE was more efficient and precise in clustering the distinct features, as compared with the commonly utilized unsupervised ordination method, PCA. In addition, the robustness and reliability of the model were confirmed in a validation set.

The central finding of our study is the identification of three distinct immunosuppression genes, PECAM1, FCGR3A, and FOS, which could be potentially high-value biomarkers or candidate therapeutic targets for periodontitis. PECAM1, also known as CD31, is an immunoglobulin (Ig) gene expressed in various cells, such as endothelial cells (ECs), platelets, and immune cells. PECAM1 is found to be a co-modulator of T-cell immunity (Huang et al., 2017) and a promoter of endothelial junctional integrity (Marelli-Berg et al., 2013). Periodontal pathogens, particularly *P. gingivalis*, can induce vascular damage through the degradation of PECAM1 (Yun et al., 2005; Farrugia et al., 2020). A protective effect of PECAM1 was also detected in transplant arteriosclerosis (Ensminger et al., 2002). FCGR3A is a member of FCGR families, forming a critical link between humoral and cellular immune responses to periodontal microbiota (Chai et al., 2010; Pavkovic et al., 2018). Previous studies have reported single-nucleotide polymorphisms (SNPs) of FCGR3A (rs396991 and rs4455090) were correlated with periodontitis and might impact susceptibility to periodontitis (Kobayashi et al., 2001; Chai et al., 2010). Besides, FCGR3A polymorphism and the allele rs396991 was identified as an independent susceptibility marker of allograft rejection in patients after organ transplants, highly responsive to natural killer (NK) cells (Paul et al., 2019). FOS was also identified as a significant TF in the study.

Of the top 10 hub TFs, six “leader” immunosuppressive TF-target DEGs with plausible literature evidence were identified as key to periodontitis pathogenesis and included the down-regulated TFs (NFKB1, FOS, and JUN), as well as up-regulated TFs (HIF1A, STAT5B, and STAT4). NFKB1, also termed NF-κB, is a core TF implicated in immune and inflammatory diseases (Tak and Firestein, 2001). Periodontal pathogens can activate NF-κB, and thus inhibition of NF-κB might be a therapeutic target for periodontitis (Ambili et al., 2005). Furthermore, NF-κB is activated in transplanted tissue, and its blockade may be potent in preventing allograft rejection after solid organ transplants, considering the role of NF-κB in T cell activation and differentiation (Molinero and Alegre, 2012). FOS is implicated in periodontitis progression acting via the regulation of T-cell receptor (TCR) signaling (Maekawa et al., 2017). C-Jun (encoded by JUN) signaling is activated by Receptor Activator of Nuclear Factor Kb Ligand (RANKL) and essential for osteoclast differentiation (Ikeda et al., 2004). Activator Protein (AP)-1 is a heterodimer composed of the Fos and Jun subunits, which downregulates osteoprotegerin and is highly expressed in periodontal ligament cells, suggesting their role in bone resorption during periodontitis (Suda et al., 2009). Inhibition of c-Fos/AP-1 by T-5224 (a novel chemical) could attenuate inflammation, T cell proliferation, and allograft rejection in pancreatic islet transplantation (PIT) (Yoshida et al., 2015) and be suggested as a target for immunosuppressive therapy. HIF1A/HIF1, an oxygen-regulated subunit (Corrado and Fontana, 2020), is involved in the immune response of periodontitis, playing a pleitropic role in defending against macrobiotics and facilitating the progression of periodontitis (Wang et al., 2017). HIF1 was also suggested to mediate inflammation and immune responses after organ transplantation, mediating angiogenesis and allograft in the donor organs (Xu et al., 2019). STAT5B and STAT4 are members of the STAT family that play important roles in activating gene transcription through various cytokines. STAT5B and STAT4 can be activated by a variety of cytokines, including Interleukin (IL)12, Type I Interferon (IFNI), IL23, IL2, IL27, and IL35 (Garcia de Aquino et al., 2009; Sanpaolo et al., 2020; Yang et al., 2020), which are prominently involved in mediating immune responses during periodontitis. IFN-γ could stimulate the expression of Indoleamine 2,3-Dioxygenase (IDO)1, a critical immunosuppression protein, in primary human periodontal ligament stem cells (Andrukhov et al., 2017). Thus, evidence suggests STAT5B and STAT4 may mediate immunosuppression during periodontitis.

The immunosuppression DEGs in the two subtypes were functionally related to multiple immune-related biological processes and pathways, and the two subtypes were distinct in their regulating pathways. In subtype S1, PD1/PLL1 checkpoint signaling, T cell receptor signaling, and signaling pathways related to immunosuppressive factors, including cytokines, chemokines, Janus Kinase (JAK) -STAT, and HIF1, are found to activate up-regulated TFs, such as HIF1A, STAT4, and STAT5B (de Souza et al., 2012). Whereas the signaling pathways enriched in subtype S2 primarily regulated the MAPK signaling pathway and osteoclast differentiation, as well as the infection of virus and *E. coli* bacteria, targeting the down-regulated TFs, such as NFKB1, FOS, and JUN (de Souza et al., 2012). Immune response-related pathways were mainly involved in the subtype S1, supporting a hypothesis that periodontitis patients with molecular subtype S1 may be more sensitive to and thus respond comparatively well to the immune-related target therapy.

Considering PDL1/PD1 signaling that characterized the subtype S1, it has been found that peptidoglycans from *P. gingivalis* can lead to the up-regulation of PDL1 expressed by gingival keratinocytes, as well as the overexpression of PD1 expressed on T lymphocytes (Bailly, 2020). The interaction between PDL1 and PD1 can suppress the initial activation and effector function of T cells and thereby promote the progression of periodontal inflammation (Yang et al., 2019). As PDL1-inhibitor has shown significant effects as a cancer therapy in clinical trials (Kim et al., 2020), it may also hold potential as immune therapy for periodontitis patients, especially in the case of immune-compromised patients. The inhibition of the JAK-STAT pathway has been indicated as a potential strategy for immunosuppression therapy, targeting the key cytokines, such as IFNg and IL12 (O’Shea and Plenge, 2012). HIF1A pathway has been found to modulate immunosuppressive molecules, typically VEGF, in periodontitis (Vasconcelos et al., 2016), and tumor microenvironment (El-Sayed Mohammed Youssef et al., 2015). Manipulation of the HIF1A pathway has been proposed as a therapeutic intervention in tumor immunotherapy (Li et al., 2018). The MAPK pathway identified in subtype S2, consists of three family sub-members, extracellular regulated kinases (ERK), c-Jun N-terminal activated kinases (JNK), and p38, and is closely related to osteoblast differentiation (Rodríguez-Carballo et al., 2016). Further, inhibition of p38 may particularly have potential therapeutic value in limiting periodontitis progression at multiple levels of the immune response via its effects on different extracellular stimuli (Kirkwood and Rossa, 2009). Of note, bone resorption, a hallmark of periodontitis, is mainly affected through RANKL, a vital osteoclast differentiation factor (Taubman et al., 2005) and Tumor Necrosis Factor (TNF)-a, majorly activated by MAPK and NF-κB pathways (Ketherin and Sandra, 2018), indicating a key role of these pathways in osteoimmunology.

Altogether, using the DL-based predictive model and bioinformatic analysis, our study provides a predictive and theoretical description of functions and mechanisms relevant to immunosuppression genes active in periodontitis pathogenesis. The validated efficiency and accuracy of the DL-model overcome the bottlenecks of current evidence and suggest new insights valuable for potential translation in therapeutic gene targeting. However, considering our study is the first to apply DL methods in the periodontal disease context, it is expected that further well-designed investigations can validate the model considering other aspects of periodontal disease, where specific and precise associations between clinical parameters and target genes might be identified. One caveat of our study is the lack of phenotype information about the periodontitis cases which were grouped into two distinct immune subtypes. Periodontitis is well recognized as a multifactorial disease, where a disease phenotype may result from multiple factors in a “sufficient cause model” (Heaton and Dietrich, 2012). Distinct “immunotypes” in periodontitis may represent heterogeneity in the core biological mechanisms contributing to disease in different subjects. A more in-depth understanding of these could support precision medicine approaches in the future. Besides, the possible clinical translation of these results may include multiple directions. For instance, the identification of immunosuppression genes may direct the development of improved topical drugs for delivery at diseased periodontal sites, which could avoid side effects inherent to conventional drugs such as antimicrobials. Also, these findings support a hypothesis that manipulation of the identified immunosuppression genes or selection of the drugs targeting immune checkpoints could be protective against periodontal diseases in patients who have had long-time immunosuppressive therapy, such as those with organ transplantation.

## Conclusion

The DL-based model applied in this study was reliable and robust in predicting immunosuppression genes in periodontitis. An array of pathways and TF-target DEGs were found to be implicated in the immunosuppressive activity during periodontitis. Three “master” immunosuppression genes, PECAM1, FCGR3A, and FOS, were identified as critical to immune suppression occurring during periodontal pathology. Taken together, the DL model revealed novel insights into the molecular mechanisms underpinning periodontitis and identified key candidate genes for further translation in the context of risk profiling and therapeutic development.

## Data Availability Statement

The original contributions presented in the study are included in the article/supplementary material, further inquiries can be directed to the corresponding authors.

## Author Contributions

WN conceived of the presented idea, designed the overall study workflow, analyzed the data, prepared the figures and tables, authored and reviewed drafts of the manuscript, and approved the final draft. AA prepared the figures and tables, was involved in the proofreading and deep editing, and approved the final draft. ZS analyzed the data, prepared the figures and tables, and was involved in the discussion of the results. AO analyzed the data, prepared the figures and tables, and was involved in proofreading and deep editing. CL, SH, QO, and MZ were involved in the discussion of the results, and also prepared the figures and tables. XL and YD designed the overall study workflow, analyzed the data, and prepared the figures and tables. RH, DZ, and GS devised the main conceptual idea, supervised the whole work, and approved the final draft. GP, YW, and XH supervised the whole project, and approved the final draft. All authors contributed to the article and approved the submitted version.

## Conflict of Interest

The authors declare that the research was conducted in the absence of any commercial or financial relationships that could be construed as a potential conflict of interest.

## Footnotes

1 http://www.ncbi.nlm.nih.gov/geo/

2 http://www.disgenet.org

3 http://biokb.ncpsb.org/HisgAtlas/

4 https://github.com/fchollet/keras

5 https://ggplot2.tidyverse.org

6 https://www.grnpedia.org/trrust/

7 https://www.scbit.org/cgrnb

8 http://www.lbbc.ibb.unesp.br/htri/

9 http://orti.sydney.edu.au/about.html

10 http://gene-regulation.com/pub/databases.html

## References

[B1] Alvarez C. Rojas C. Rojas L. Cafferata E. A. Monasterio G. Vernal R. (2018). Regulatory T lymphocytes in periodontitis: a translational view. *Mediators Inflamm.* 2018:7806912. 10.1155/2018/7806912 29805313 PMC5901475

[B2] Ambili R. Santhi W. S. Janam P. Nandakumar K. Pillai M. R. (2005). Expression of activated transcription factor nuclear factor-kappaB in periodontally diseased tissues. *J. Periodontol.* 76 1148–1153. 16018758 10.1902/jop.2005.76.7.1148

[B3] Andrukhov O. Hong J. S. A. Andrukhova O. Blufstein A. Moritz A. Rausch-Fan X. (2017). Response of human periodontal ligament stem cells to IFN-γ and TLR-agonists. *Sci. Rep.* 7:12856. 10.1038/s41598-017-12480-7 28993635 PMC5634407

[B4] Aoyagi T. Yamazaki K. Kabasawa-Katoh Y. Nakajima T. Yamashita N. Yoshie H. (2000). Elevated CTLA-4 expression on CD4 T cells from periodontitis patients stimulated with *Porphyromonas gingivalis* outer membrane antigen: CTLA-4 expression in periodontitis. *Clin. Exp. Immunol.* 119 280–286. 10.1046/j.1365-2249.2000.01126.x 10632663 PMC1905507

[B5] Bailly C. (2020). The implication of the PD-1/PD-L1 checkpoint in chronic periodontitis suggests novel therapeutic opportunities with natural products. *Jpn. Dent. Sci Rev.* 56 90–96. 10.1016/j.jdsr.2020.04.002 32612718 PMC7310691

[B6] Calinski T. Harabasz J. (1974). A dendrite method for cluster analysis. *Commun. Stat. Theory Methods* 3 1–27. 10.1080/03610927408827101

[B7] Cebeci I. Kantarci A. Firatli E. Aygun S. Tanyeri H. Aydin A. E. (1996). Evaluation of the frequency of HLA determinants in patients with gingival overgrowth induced by cyclosporine-A. *J. Clin. Periodontol.* 23 737–742. 10.1111/j.1600-051X.1996.tb00603.x 8877659

[B8] Cekici A. Kantarci A. Hasturk H. Van Dyke T. E. (2014). Inflammatory and immune pathways in the pathogenesis of periodontal disease: inflammatory and immune pathways in periodontal disease. *Periodontol. 2000* 64 57–80. 10.1111/prd.12002 24320956 PMC4500791

[B9] Chai L. Song Y.-Q. Zee K.-Y. Leung W. K. (2010). SNPs of Fc-gamma receptor genes and chronic periodontitis. *J. Dent. Res.* 89 705–710. 10.1177/0022034510365444 20439936

[B10] Chaudhary K. Poirion O. B. Lu L. Garmire L. X. (2018). Deep learning–based multi-omics integration robustly predicts survival in liver cancer. *Clin. Cancer Res.* 24 1248–1259. 10.1158/1078-0432.CCR-17-0853 28982688 PMC6050171

[B11] Chicco D. Sadowski P. Baldi P. (2014). “Deep autoencoder neural networks for gene ontology annotation predictions,” in *Proceedings of the 5th ACM Conference on Bioinformatics, Computational Biology, and Health Informatics*, (Newport Beach, CA: ACM), 533–540. 10.1145/2649387.2649442

[B12] Corrado C. Fontana S. (2020). Hypoxia and HIF signaling: one axis with divergent effects. *Int. J. Mol. Sci.* 21:5611. 10.3390/ijms21165611 32764403 PMC7460602

[B13] Cota L. O. M. Aquino D. R. Franco G. C. N. Cortelli J. R. Cortelli S. C. Costa F. O. (2010). Gingival overgrowth in subjects under immunosuppressive regimens based on cyclosporine, tacrolimus, or sirolimus: risk variables for gingival overgrowth. *J. Clin. Periodontol.* 37 894–902. 10.1111/j.1600-051X.2010.01601.x 20618547

[B14] de Souza J. A. C. Junior C. R. Garlet G. P. Nogueira A. V. B. Cirelli J. A. (2012). Modulation of host cell signaling pathways as a therapeutic approach in periodontal disease. *J. Appl. Oral Sci.* 20 128–138. 10.1590/S1678-77572012000200002 22666826 PMC3894752

[B15] Dutzan N. Konkel J. E. Greenwell-Wild T. Moutsopoulos N. M. (2016). Characterization of the human immune cell network at the gingival barrier. *Mucosal. Immunol.* 9 1163–1172. 10.1038/mi.2015.136 26732676 PMC4820049

[B16] El-Sayed Mohammed Youssef H. Eldeen Abo-Azma N. E. Eldeen Megahed E. M. (2015). Correlation of hypoxia-inducible factor-1 alpha (HIF-1α) and vascular endothelial growth factor (VEGF) expressions with clinico-pathological features of oral squamous cell carcinoma (OSCC). *Tanta Dent. J.* 12 S1–S14. 10.1016/j.tdj.2015.05.010

[B17] Ensminger S. M. Spriewald B. M. Steger U. Morris P. J. Mak T. W. Wood K. J. (2002). Platelet-endothelial cell adhesion molecule-1 (CD31) expression on donor endothelial cells attenuates the development of transplant arteriosclerosis. *Transplantation* 74 1267–1273. 10.1097/00007890-200211150-00012 12451264

[B18] Farrugia C. Stafford G. P. Potempa J. Wilkinson R. N. Chen Y. Murdoch C. (2020). Mechanisms of vascular damage by systemic dissemination of the oral pathogen *Porphyromonas gingivalis*. *FEBS J.* 15486. 10.1111/febs.15486 32681704 PMC9994420

[B19] Galili T. O’Callaghan A. Sidi J. Sievert C. (2017). heatmaply: an R package for creating interactive cluster heatmaps for online publishing. *Bioinformatics* 34 1600–1602. 10.1093/bioinformatics/btx657 29069305 PMC5925766

[B20] Garcia de Aquino S. Manzolli Leite F. R. Stach-Machado D. R. Francisco da Silva J. A. Spolidorio L. C. Rossa C. (2009). Signaling pathways associated with the expression of inflammatory mediators activated during the course of two models of experimental periodontitis. *Life Sci.* 84 745–754. 10.1016/j.lfs.2009.03.00 19285515

[B21] Gemmell E. Yamazaki K. Seymour G. J. (2002). Destructive periodontitis lesions are determined by the nature of the lymphocytic response. *Crit. Rev. Oral Biol. Med.* 13 17–34. 10.1177/154411130201300104 12097235

[B22] Heaton B. Dietrich T. (2012). Causal theory and the etiology of periodontal diseases. *Periodontol.* 2000 26–36. 10.1111/j.1600-0757.2011 22133365

[B23] Huang F. Chen M. Chen W. Gu J. Yuan J. Xue Y. (2017). Human gingiva-derived mesenchymal stem cells inhibit xeno-graft-versus-host disease via CD39–CD73–adenosine and IDO signals. *Front. Immunol.* 8:68. 10.3389/fimmu.2017.00068 28210258 PMC5288353

[B24] Ikeda F. Nishimura R. Matsubara T. Tanaka S. Inoue J. Reddy S. V. (2004). Critical roles of c-Jun signaling in regulation of NFAT family and RANKL-regulated osteoclast differentiation. *J. Clin. Invest.* 114 475–484. 10.1172/JCI200419657 15314684 PMC503767

[B25] Ju Y. Guo J. Liu S. (2015). “A deep learning method combined sparse autoencoder with SVM,” in *Proceedings of the 2015 International Conference on Cyber-Enabled Distributed Computing and Knowledge Discovery*, (Xi’an: IEEE), 257–260. 10.1109/CyberC.2015.39

[B26] Ketherin K. Sandra F. (2018). Osteoclastogenesis in periodontitis: signaling pathway. Synthetic and natural inhibitors. *Mol. Cell. Biomed. Sci.* 2:11. 10.21705/mcbs.v2i1.16

[B27] Kim H. Kwon M. Kim B. Jung H. A. Sun J.-M. Lee S.-H. (2020). Clinical outcomes of immune checkpoint inhibitors for patients with recurrent or metastatic head and neck cancer: real-world data in Korea. *BMC Cancer* 20:727. 10.1186/s12885-020-07214-4 32758163 PMC7405432

[B28] Kirkwood K. L. Rossa C. Jr. (2009). The potential of p38 MAPK inhibitors to modulate periodontal infections. *Curr. Drug Metab.* 10 55–67. 10.2174/138920009787048347 19149513 PMC2810486

[B29] Kobayashi T. Yamamoto K. Sugita N. van der Pol W. L. Yasuda K. Kaneko S. (2001). The Fc gamma receptor genotype as a severity factor for chronic periodontitis in Japanese patients. *J. Periodontol.* 72 1324–1331. 10.1902/jop.2001.72.10.1324 11699473

[B30] Lee H. Grosse R. Ranganath R. Ng A. Y. (2009). “Convolutional deep belief networks for scalable unsupervised learning of hierarchical representations,” in *Proceedings of the 26th Annual International Conference on Machine Learning – ICML ‘09*, (Montreal, QC: ACM Press), 1–8. 10.1145/1553374.1553453

[B31] Li Y. Patel S. P. Roszik J. Qin Y. (2018). Hypoxia-Driven immunosuppressive metabolites in the tumor microenvironment: new approaches for combinational immunotherapy. *Front. Immunol.* 9:1591. 10.3389/fimmu.2018.01591 30061885 PMC6054965

[B32] Maekawa T. Kulwattanaporn P. Hosur K. Domon H. Oda M. Terao Y. (2017). Differential expression and roles of secreted frizzled-related protein 5 and the wingless homolog Wnt5a in periodontitis. *J. Dent. Res.* 96 571–577. 10.1177/0022034516687248 28095260 PMC5453495

[B33] Mandrekar J. N. (2010). Receiver operating characteristic curve in diagnostic test assessment. *J. Thorac. Oncol.* 5 1315–1316. 10.1097/jto.0b013e3181ec173d 20736804

[B34] Marelli-Berg F. M. Clement M. Mauro C. Caligiuri G. (2013). An immunologist’s guide to CD31 function in T-cells. *J. Cell Sci.* 126 2343–2352. 10.1242/jcs.124099 23761922

[B35] Meyle J. Chapple I. (2015). Molecular aspects of the pathogenesis of periodontitis. *Periodontol. 2000* 69 7–17. 10.1111/prd.12104 26252398

[B36] Molinero L. L. Alegre M.-L. (2012). Role of T cell–nuclear factor κB in transplantation. *Transplant. Rev.* 26 189–200. 10.1016/j.trre.2011.07.005 22074783 PMC3309102

[B37] O’Shea J. J. Plenge R. (2012). JAK and STAT signaling molecules in immunoregulation and immune-mediated disease. *Immunity* 36 542–550. 10.1016/j.immuni.2012.03.014 22520847 PMC3499974

[B38] Paul P. Pedini P. Lyonnet L. Di Cristofaro J. Loundou A. Pelardy M. (2019). FCGR3A and FCGR2A genotypes differentially impact allograft rejection and patients’ survival after lung transplant. *Front. Immunol.* 10:1208. 10.3389/fimmu.2019.01208 31249568 PMC6582937

[B39] Pavkovic M. Petlichkovski A. Karanfilski O. Cevreska L. Stojanovic A. (2018). FC gamma receptor polymorphisms in patients with immune thrombocytopenia. *Hematology* 23 163–168. 10.1080/10245332.2017.1377902 28942727

[B40] Pedregosa F. Varoquaux G. Gramfort A. Michel V. Thirion B. Grisel O. (2011). Scikit-learn: machine learning in python. *J. Mach. Learn. Res.* 12 2825–2830.

[B41] Ritchie M. E. Phipson B. Wu D. Hu Y. Law C. W. Shi W. (2015). Limma powers differential expression analyses for RNA-sequencing and microarray studies. *Nucleic Acids Res.* 43:e47. 10.1093/nar/gkv007 25605792 PMC4402510

[B42] Robin X. Turck N. Hainard A. Tiberti N. Lisacek F. Sanchez J. (2011). pROC: an open-source package for R and S+ to analyze and compare ROC curves. *BMC Bioinformatics* 12:77. 10.1186/1471-2105-12-77 21414208 PMC3068975

[B43] Rodríguez-Carballo E. Gámez B. Ventura F. (2016). p38 MAPK signaling in osteoblast differentiation. *Front. Cell. Dev. Biol.* 4:40. 10.3389/fcell.2016.00040 27200351 PMC4858538

[B44] Rousseeuw P. J. (1987). Silhouettes: a graphical aid to the interpretation and validation of cluster analysis. *J. Comput. Appl. Math.* 20 53–65. 10.1016/0377-0427(87)90125-7

[B45] Sanpaolo E.R. Rotondo C. Cici D. Corrado A. Cantatore F.P. (2020). JAK/STAT pathway and molecular mechanism in bone remodeling. *Mol. Biol. Rep.* 47 9087–9096. 10.1007/s11033-020-05910-9 33099760 PMC7674338

[B46] Scott D. A. Krauss J. (2011). “Neutrophils in periodontal inflammation,” in *Frontiers of Oral Biology*, eds Kinane D. F. Mombelli A. (Basel: KARGER), 56–83. 10.1159/000329672 PMC3335266 22142957

[B47] Shannon P. Markiel A. Ozier O. Baliga N. S. Wang J. T. Ramage D. (2003). Cytoscape: a software environment for integrated models of biomolecular interaction networks. *Genome Res.* 13 2498–2504. 10.1101/gr.1239303 14597658 PMC403769

[B48] Suda T. Nagasawa T. Wara-aswapati N. Kobayashi H. Iwasaki K. Yashiro R. (2009). Regulatory roles of β-catenin and AP-1 on osteoprotegerin production in interleukin-1α-stimulated periodontal ligament cells. *Oral Microbiol. Immunol.* 24 384–389. 10.1111/j.1399-302X.2009.00529.x 19702951

[B49] Tak P. P. Firestein G. S. (2001). NF-κB: a key role in inflammatory diseases. *J. Clin. Invest.* 107 7–11. 10.1172/jci11830 11134171 PMC198552

[B50] Tan J. Ung M. Cheng C. Greene C. S. (2014). “Unsupervised feature construction and knowledge extraction from genome-wide assays of breast cancer with denoising autoencoders,” in *Biocomputing 2015*, (Kohala Coast, HI: World Scientific), 132–143. 10.1142/9789814644730_0014 PMC4299935 25592575

[B51] Taubman M. A. Valverde P. Han X. Kawai T. (2005). Immune response: the key to bone resorption in periodontal disease. *J. Periodontol.* 76 2033–2041. 10.1902/jop.2005.76.11-s.2033 16277573

[B52] Vasconcelos R. C. Costa A. D. L. L. Freitas R. D. A. Bezerra B. A. D. A. Santos B. R. M. D. Pinto L. P. (2016). Immunoexpression of HIF-1α and VEGF in periodontal disease and healthy gingival tissues. *Braz. Dent. J.* 27 117–122. 10.1590/0103-6440201600533 27058371

[B53] Wang X. X. Chen Y. Leung W. K. (2017). “Role of the hypoxia-inducible factor in periodontal inflammation,” in *Hypoxia and Human Diseases*, eds Zheng J. Zhou C. (Rijeka: Intech Open), 285–302.

[B54] Wang Y. Yao H. Zhao S. (2016). Auto-encoder based dimensionality reduction. *Neurocomputing* 184 232–242. 10.1016/j.neucom.2015.08.104

[B55] Xiao Y. Wu J. Lin Z. Zhao X. (2018). A semi-supervised deep learning method based on stacked sparse auto-encoder for cancer prediction using RNA-seq data. *Comput. Methods Programs Biomed.* 166 99–105. 10.1016/j.cmpb.2018.10.004 30415723

[B56] Xu H. Abuduwufuer A. Lv W. Zhou Z. Yang Y. Zhang C. (2019). The role of HIF-1α-VEGF pathway in bronchiolitis obliterans after lung transplantation. *J. Cardiothorac. Surg.* 14:27. 10.1186/s13019-019-0832-z 30696477 PMC6352448

[B57] Yang C. Mai H. Peng J. Zhou B. Hou J. Jiang D. (2020). STAT4: an immunoregulator contributing to diverse human diseases. *Int. J. Biol. Sci.* 16 1575–1585. 10.7150/ijbs.41852 32226303 PMC7097918

[B58] Yang X. Yang X. H. Zhang W. Z. (2019). Temporal expression of PD-1 and PD-L1 during the development of experimental periodontitis in rats and its implications. *Shanghai Kou Qiang Yi Xue* 28 591–596. Chinese. 32346701

[B59] Yoshida T. Yamashita K. Watanabe M. Koshizuka Y. Kuraya D. Ogura M. (2015). The impact of c-Fos/Activator protein-1 inhibition on allogeneic pancreatic Islet transplantation. *Am. J. Transplant.* 15 2565–2575. 10.1111/ajt.13338 26012352

[B60] Yu G. Wang L. Han Y. He Q. (2012). ClusterProfiler: an R package for comparing biological themes among gene clusters. *OMICS* 16 284–287. 10.1089/omi.2011.0118 22455463 PMC3339379

[B61] Yun P. L. W. Decarlo A. A. Chapple C. C. Hunter N. (2005). Functional implication of the hydrolysis of platelet endothelial cell adhesion molecule 1 (CD31) by gingipains of *Porphyromonas gingivalis* for the pathology of periodontal disease. *Infect. Immun.* 73 1386–1398. 10.1128/IAI.73.3.1386-1398.2005 15731036 PMC1064963

[B62] Zhao Z. Li Y. Wu Y. Chen R. (2019). Deep learning-based model for predicting progression in patients with head and neck squamous cell carcinoma. *Cancer Biomark.* 27 19–28. 10.3233/CBM-190380 31658045 PMC12662277

[B63] Zhou L. Bi C. Gao L. An Y. Chen F. Chen F. (2019). Macrophag1996e polarization in human gingival tissue in response to periodontal disease. *Oral Dis.* 25 265–273. 10.1111/odi.12983 30285304

